# Risk prediction of ischemic heart disease using plasma proteomics, conventional risk factors and polygenic scores in Chinese and European adults

**DOI:** 10.1007/s10654-024-01168-8

**Published:** 2024-11-22

**Authors:** Mohsen Mazidi, Neil Wright, Pang Yao, Christiana Kartsonaki, Iona Y. Millwood, Hannah Fry, Saredo Said, Alfred Pozarickij, Pei Pei, Yiping Chen, Baihan Wang, Daniel Avery, Huaidong Du, Dan Valle Schmidt, Ling Yang, Jun Lv, Canqing Yu, DianJianYi Sun, Junshi Chen, Michael Hill, Richard Peto, Rory Collins, Derrick A. Bennett, Robin G. Walters, Liming Li, Robert Clarke, Zhengming Chen, China Kadoorie Biobank Collaborative Group

**Affiliations:** 1 Clinical Trial Service Unit, Nuffield Department of Population Health, University of Oxford, Old Road Campus, Roosevelt Drive, Oxford, OX3 7LF UK; 2 Department of Epidemiology and Biostatistics, School of Public Health, Peking University Health Science Center, Beijing, China; 3 Center for Public Health and Epidemic Preparedness and Response, Peking University, Beijing, China; 4 Key Laboratory of Epidemiology of Major (Peking University), Ministry of Education, Beijing, China; 5 China National Center for Food Risk Assessment, Beijing, China

**Keywords:** Plasma proteomics, Risk prediction, Polygenic score, Ischaemic heart disease, Prospective studies, Machine learning

## Abstract

Plasma proteomics could enhance risk prediction for multiple diseases beyond conventional risk factors or polygenic scores (PS). To assess utility of proteomics for risk prediction of ischemic heart disease (IHD) compared with conventional risk factors and PS in Chinese and European populations. A nested case-cohort study measured plasma levels of 2923 proteins using Olink Explore panel in ~ 4000 Chinese adults (1976 incident IHD cases and 2001 sub-cohort controls). We used conventional and machine learning (Boruta) methods to develop proteomics-based prediction models of IHD, with discrimination assessed using area under the curve (AUC), C-statistics and net reclassification index (NRI). These were compared with conventional risk factors and PS in Chinese and in 37,187 Europeans. Overall, 446 proteins were associated with IHD (false discovery rate < 0.05) in Chinese after adjustment for conventional cardiovascular disease risk factors. Proteomic risk models alone yielded higher C-statistics for IHD than conventional risk factors or PS (0.855 [95%CI 0.841–0.868] vs. 0.845 [0.829–0.860] vs 0.553 [0.528–0.578], respectively). Addition of 446 proteins to PS improved C-statistics to 0.857 (0.843–0.871) and NRI by 109.1%; and addition to conventional risk factors improved C-statistics to 0.868 (0.854–0.882) and NRI by 86.9%. Boruta analysis identified 30 proteins accounting for ~ 90% of improvement in NRI for IHD conferred by all 2923 proteins. Similar proteomic panels yielded comparable improvements in risk prediction of IHD in Europeans. Plasma proteomics improved risk prediction of IHD beyond conventional risk factors and PS and could enhance precision medicine approaches for primary prevention of IHD.

**Supplementary Information:**

The online version contains supplementary material available at 10.1007/s10654-024-01168-8.

## Introduction

Despite recent improvements in prevention and treatment, ischemic heart disease (IHD) remains a major cause of premature death worldwide, with an increasing prevalence in low and middle-income countries including China [1]. Conventional modifiable risk factors for IHD (e.g. hypertension, dyslipidaemia, and cigarette smoking) are widely used to predict risk of IHD to optimize prevention and treatment of IHD [2]. Recent genome-wide association studies identified a large number of genetic variants associated with IHD in diverse ancestry populations, prompting the development and use of polygenic scores (PS) either alone or in combination with conventional risk models (e.g., SCORE2 or Pooled Cohorts Equations [PCE]) to improve risk prediction of IHD [3]. Several studies have suggested that PS for IHD could identify high-risk individuals for more intensive lifestyle modification, imaging surveillance and early statin therapy. [4]

Previous prospective studies have reported that the addition of individual proteins including C-reactive protein, interleukin-6, fibrinogen, N-terminal pro B-type natriuretic peptide (NT-proBNP) or lipoprotein (a) to conventional risk factors also yielded modest improvement in prediction of IHD risk [5]. Advances in high-throughput proteomic assays now enable reliable quantification of plasma levels of thousands of proteins [6]. Recently, several studies have evaluated the utility of plasma proteomics for risk prediction of cardiovascular disease (CVD) and demonstrated improvements in risk prediction beyond conventional risk models [7–13]. However, most such studies have been constrained by small numbers of participants, limited numbers of proteins, failure to account for effects of conventional CVD risk factors, or lack of external validation in independent populations [7–9, 13]. Moreover, previous studies have been restricted to European ancestry populations, with little data from other ancestry populations where disease rates, distribution of risk factors or genetic architecture differ from European ancestry populations [14]. Furthermore, no previous studies have systematically compared the separate or combined effects of plasma proteomics with conventional and PS for prediction of IHD.

Using proteomic and genomic data in the prospective China Kadoorie Biobank (CKB), the present study aimed to: (i) examine the associations of 2923 proteins with risk of incident IHD independent of conventional CVD risk factors; (ii) compare risk prediction of IHD associated with plasma proteomics, PS or conventional risk factors, alone or in combination; and (iii) conduct external validation of proteomics, PS and conventional risk factors for risk prediction of IHD in European adults in the UK Biobank (UKB).

## Methods

### Study population

Details of the CKB study design, methods and participants have been previously reported. In brief, CKB recruited > 512,000 adults aged 30–79 years from 10 diverse (5 urban, 5 rural) regions in China during 2004–08. The baseline survey included a laptop-based questionnaire that collected data on socio-demographic factors, lifestyle (e.g. smoking, alcohol drinking, physical activity), medical history and medication use (e.g. statins, insulin), and physical measurements (e.g. height, weight, waist circumference, blood pressure, and lung function). Each participant also provided a 10 mL non-fasting blood sample (with time since last meal recorded) for long-term storage in liquid nitrogen. Ethical approval was granted and maintained by the relevant institutional ethical research committees in the UK (Oxford Tropical Research Ethics Committee and China. All study participants provided written informed consent.

### Follow-up for fatal and non-fatal disease outcomes

After enrolment, the vital status and occurrence of specific diseases among participants was monitored by linkage, using unique national identification numbers, with local mortality (cause-specific) and morbidity (IHD, stroke, cancer and diabetes) registers and to the National Health Insurance system (which has > 98% coverage in study regions) for any episodes of hospitalisation. All causes of death or incident diseases were international classification of diseases-10 (ICD-10) coded by trained health workers, blinded to baseline information, and checked and integrated centrally. [15]

### Case-cohort study of IHD

The present study used a case-cohort design involving 1976 incident cases of IHD (ICD-10 codes: I20-I25) that were accrued during a 12-year follow-up prior to 1 January 2019 and a sub-cohort of 2001 participants. All IHD cases and sub-cohort participants had no prior history of CVD or statin use at baseline. The IHD cases were a random sample of incident IHD cases with genome-wide association studies data, while the sub-cohort participants were randomly selected from a population subset of 69,353 genotyped participants, but were genetically unrelated to each other. [16]

### Proteomics assays

Plasma levels of 2923 proteins were measured using the Olink Explore 3072 panel in two independent batches, with 1st Batch (1463 proteins) assayed at the Olink laboratory in Uppsala, Sweden and 2nd Batch (1460 proteins) assayed at the Olink laboratory in Boston, USA. Data from each batch were obtained from all participants and were treated independently (Supplementary methods). The results of plasma proteomics were provided using Normalized Protein eXpression (NPX) units on logarithmic (Log 2) scale. [17]

### Statistical analyses

NPX data were standardized (i.e. with values divided by their standard deviations (SDs) and analysed as continuous variables. In observational analyses, weighted Cox regression models were used to estimate the adjusted hazard ratios (HRs) (and 95% CIs) for IHD associated with individual proteins using the Prentice pseudo-partial likelihood for case-cohort studies [18]. The sub-cohort participants who developed incident IHD during follow-up were censored at the time of diagnosis. All analyses were stratified by sex and area (10 study areas), and adjusted initially (*Model 1*) for age, age [2], time since last meal and its square, ambient temperature and its square, and plate ID*,* followed by additional sequential adjustments for (i) education (five categories) (*Model 2)*; (ii) physical activity (Metabolic Equivalent Of Task-hours/day) (*Model 3*); (iii) alcohol consumption (six categories) (*Model 4*); (iv) smoking (four categories) (*Model 5*); (v) systolic blood pressure (SBP) (*Model 6*); (vi) type-2 diabetes (T2D) (yes/no) (*Model 7*); and (vii) body mass index (BMI) (*Model 8)*. For proteins significantly associated with IHD in *Model 8*, we further examined the shape of the associations with IHD by quartiles of individual proteins.

Three proteomic-based risk models for IHD were constructed using: (i) all proteins assayed; (ii) proteins significantly associated with risk of IHD in *Model 8*; and (iii) a subset of proteins identified in *Model 8* using a Boruta machine learning algorithm as being most strongly associated with risk of IHD. The Boruta classification algorithm uses importance measures from multiple runs of a random forest classification algorithm to select relevant proteins for risk prediction [19]. The selection of proteins associated with IHD in CKB was validated using tenfold cross-validation (with 90% for training and 10% for test datasets on successive iterations). Additional details on construction of the PS in CKB and model optimization are provided in the Supplementary Methods. All p-values were adjusted for multiple testing by controlling the false discovery rate (FDR) otherwise mentioned. [20] All analyses were performed used R version 4.2.2.

### External validation in UKB

We used comparable conventional risk factors and proteomics panels associated with IHD in Chinese for prediction of IHD in European participants in UKB. We then compared the proteomic model performance between the two populations. The reporting of the multivariable risk prediction models evaluated in this report followed the transparent reporting of a multivariable prediction model for individual prognosis or diagnosis (TRIPOD) guidelines for reporting risk prediction studies. [21, 22]

## Results

**Fig. 1:**
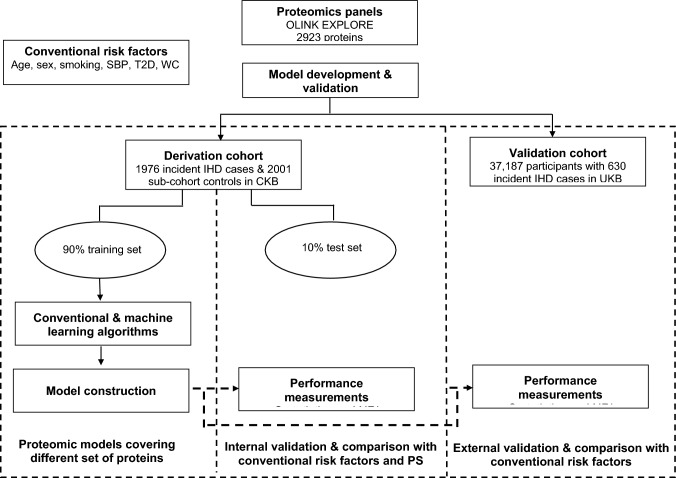
Study design and analytical approaches for discovery of an optimum proteomics panel for prediction of risk of IHD in Chinese and European adults

Overall, IHD cases in CKB were older than sub-cohort participants (mean baseline age 63.8 [SD 9.2] vs 51.2 [10.3] years), more likely to be men than women (62.1% vs 37.9%), less well-educated and current cigarette smokers. IHD cases also had a higher prevalence of hypertension and diabetes and higher mean levels of SBP, adiposity and random blood glucose than the sub-cohort participants (eTable 1). The median time from enrolment to IHD diagnosis was 6.8 years (interquartile range 4.5 years). The main analytical approaches used to select the optimum proteomics panel for risk prediction of IHD in Chinese and European adults are summarised in Fig. 1.

### Associations of proteins with risk of IHD

After basic adjustment (i.e. Model 1), a total of 584 proteins were significantly associated at FDR < 0.05 with risk of IHD (563 positively, 21 inversely), including 503 (9 inverse) in the first and 81 (12 inverse) in the second batch (Table 1). The number of proteins significantly associated with IHD increased with incremental adjustment for education and lifestyle factors (e.g. 653 in Model 5), but decreased with further additional adjustment for SBP, diabetes and BMI, resulting in 446 proteins (1st / 2nd batch: 361/85) significantly associated with IHD in the final model (i.e. Model 8), with all but 24 proteins showing positive associations with IHD risk. These 446 proteins were only moderately correlated with each other, with 99.8% and 99.7% of protein pairs having correlation coefficients in the range of -0.5 to + 0.5, respectively.

**Table 1:** Number of proteins significantly associated at FDR < 0.05 with risk of IHD after step-wise adjustment for conventional CVD risk factors in observational analyses of CKB participants, by OLINK batch

Models	OLINK Explore panel		
	1st batch (n = 1463)	2nd batch (n = 1460)	Both
	All (Inverse)	All (Inverse)	All (Inverse)
Basic model*	503 (9)	81 (12)	584 (21)
+ Education	552 (9)	101 (14)	653 (23)
+ Physical activity	537 (8)	97 (8)	633 (16)
+ Alcohol intake	538 (10)	98 (8)	636 (18)
+ Smoking	550 (10)	103 (11)	653 (21)
+ SBP	393 (10)	89 (11)	482 (21)
+ Diabetes	355 (11)	85 (12)	440 (23)
+ BMI	361 (12)	85 (12)	446 (24)

FDR = False discovery rate; CVD = Cardiovascular disease; SBP = Systolic blood pressure; BMI = Body mass index

*Basic model: Age, age^2^, fasting time, fasting time^2^, ambient temperature, ambient temperature^2^ and plate ID

**Fig. 2:**
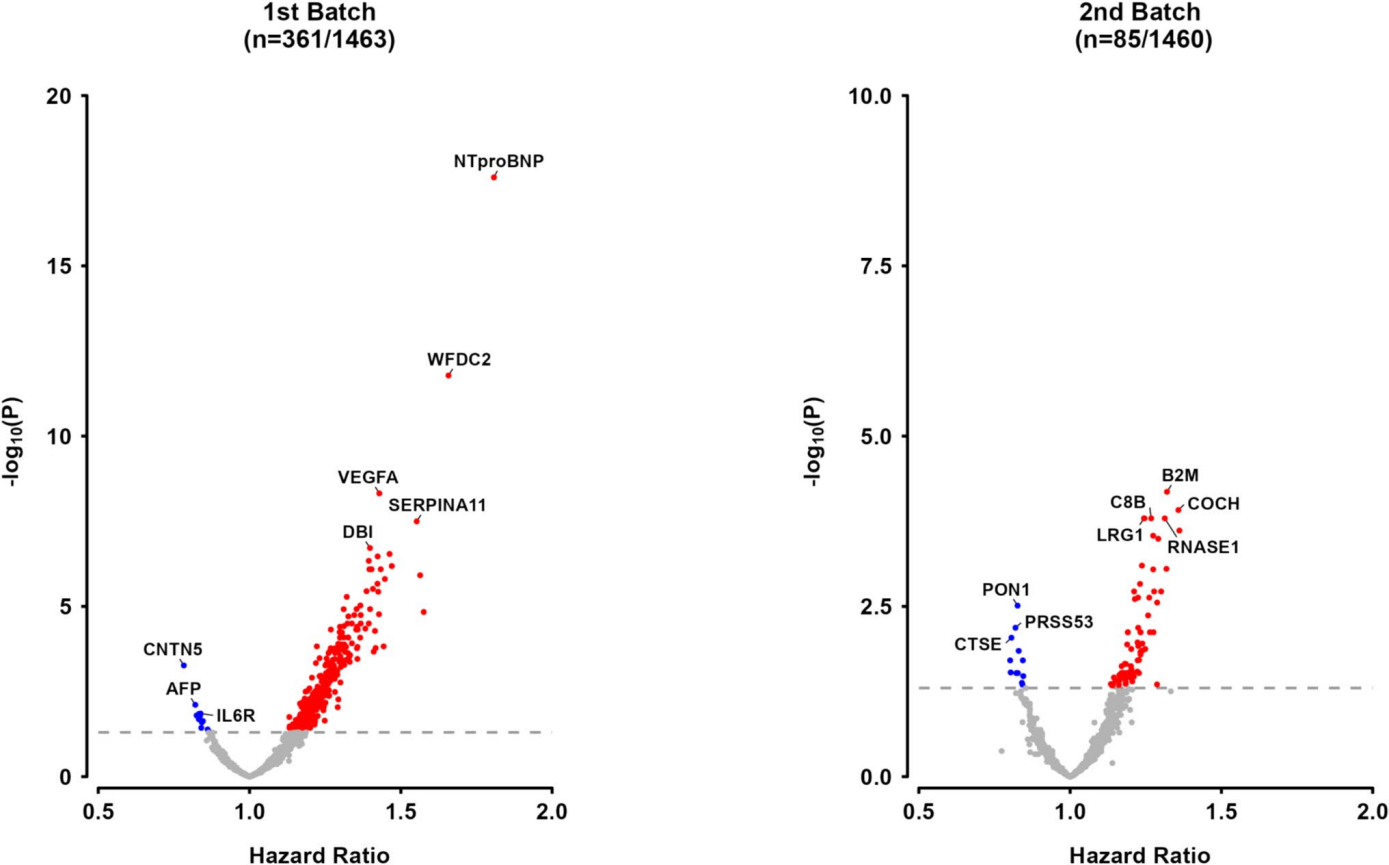
Adjusted HRs for risk of IHD associated with 1 SD higher levels of proteins in observational analyses of CKB participants, by Olink batch. Volcano plots show the association of proteins with IHD stratified by Olink panels in CKB. The values shown are FDR-adjusted p-values for individual proteins. Models were stratified by sex and region and adjusted for age, age^2^, fasting time, fasting time^2^, ambient temperature, ambient temperature^2^, plate ID, education, smoking, alcohol consumption, physical activity, SBP, type 2 diabetes, and BMI. Red, blue and grey dots denote significant positive, significant inverse and non-significant associations, respectively. Time in study was used as the time scale in the models

**Fig. 3:**
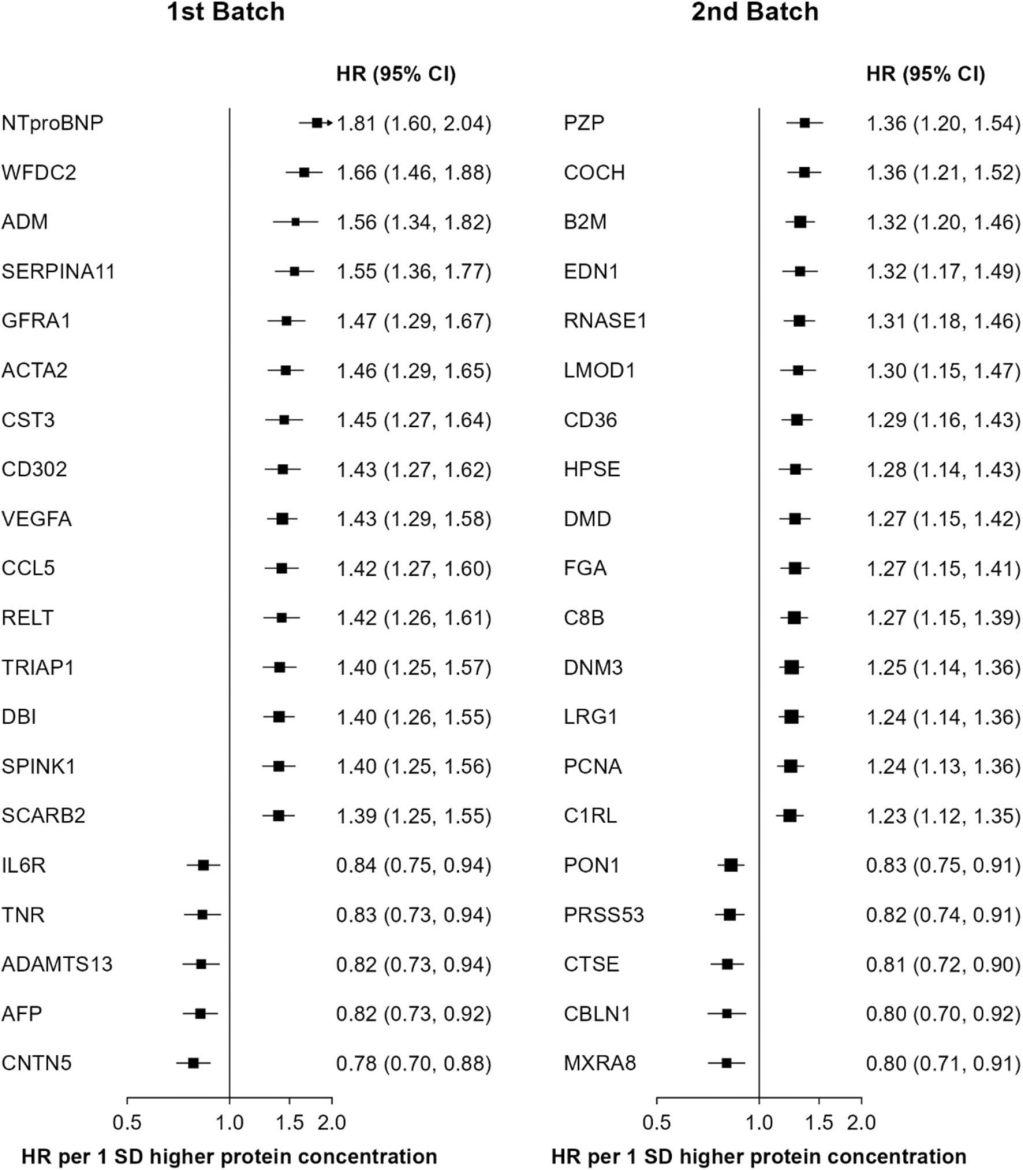
Adjusted HRs for risk of IHD associated with 1 SD higher levels of selected top 25 proteins in observational analyses of CKB participants, by Olink batch. All models were adjusted for covariates as in Fig. 1. The boxes are HRs and the horizontal lines are 95% CIs. The area of each box is inversely proportional to the variance of the log HR

Overall, these 446 proteins showed approximately log-linear associations with IHD risk, with no evidence of any threshold beyond which the levels of specific proteins were no longer associated with IHD risk (eFigure 1). In the 1st batch, the top three proteins most strongly and positively associated with IHD were NTproBNP (adjusted HR: 1.81, 95%CI 1.60–2.04, per 1 SD higher concentration), WFDC2 (1.66, 1.46–1.88) and ADM (1.56, 1.34–1.82), while the top two proteins most strongly inversely associated with IHD were CNTN5 (0.78, 0.70–0.88) and AFP (0.82, 0.73–0.92: Fig. 2, Fig. 3). In the 2nd batch, PZP (1.36, 1.20–1.54), COCH (1.36, 1.21–1.52) and B2M (1.32, 1.20–0.46) were the top three positively associated proteins with IHD, while MXRA8 (0.80, 0.71–0.91) and CBLN1 (0.80, 0.72–0.92) were the top two inversely associated proteins with IHD. The results of sensitivity analyses after exclusion of proteins with assay warnings or values below the limit of detection did not differ from the overall analyses (eTable 4).

### Prediction of risk of IHD

**Fig. 4:**
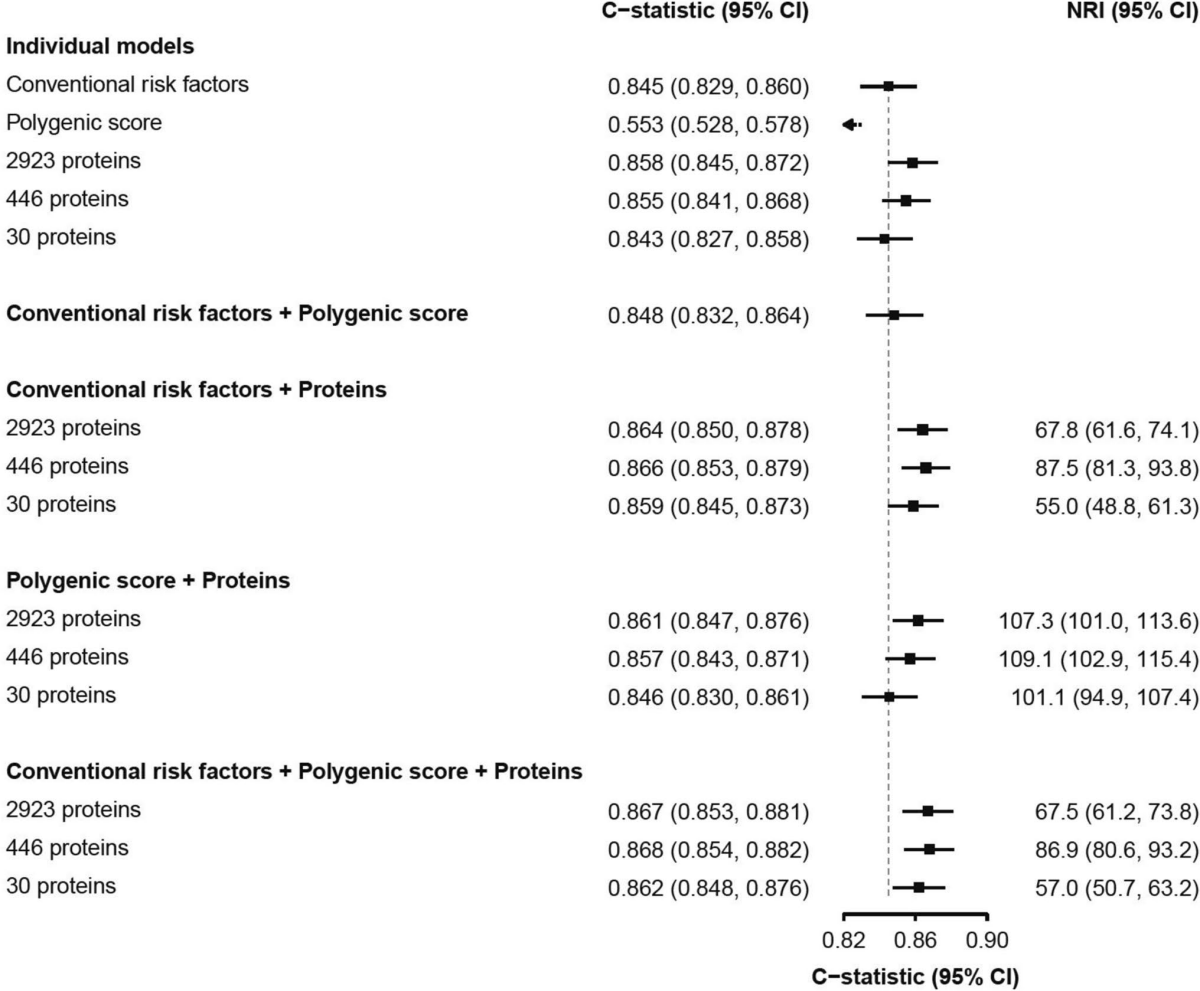
Comparison of predictive ability of conventional CVD risk factors, polygenic scores and plasma proteins for discrimination of IHD in Chinese adults. Conventional CVD risk factors include age, sex, smoking, T2D, SBP and WC

In risk prediction analyses, models using the 446 proteins significantly associated with IHD yielded C-statistics of 0.855 (95% CI 0.841–0.868) for IHD vs 0.845 (0.829–0.860) for conventional risk factors and 0.553 (0.528–0.578) for PS when each model was considered alone (Fig. 4). Additional details of the individual proteins associated with IHD in the different models are provided in the Supplement. The C-statistics for IHD were comparable for all 2923 proteins and a subset of just 30 proteins identified by Boruta (0.858 [95%CI: 0.845–0.872] vs 0.843 [0.827–0.858], respectively). The addition of apolipoprotein B to apolipoprotein A (ApoB/ApoA) ratio to conventional CVD risk factors did not alter the C-statistics for IHD (0.846 [95%CI: 0.831–0.859]).

**Fig. 5:**
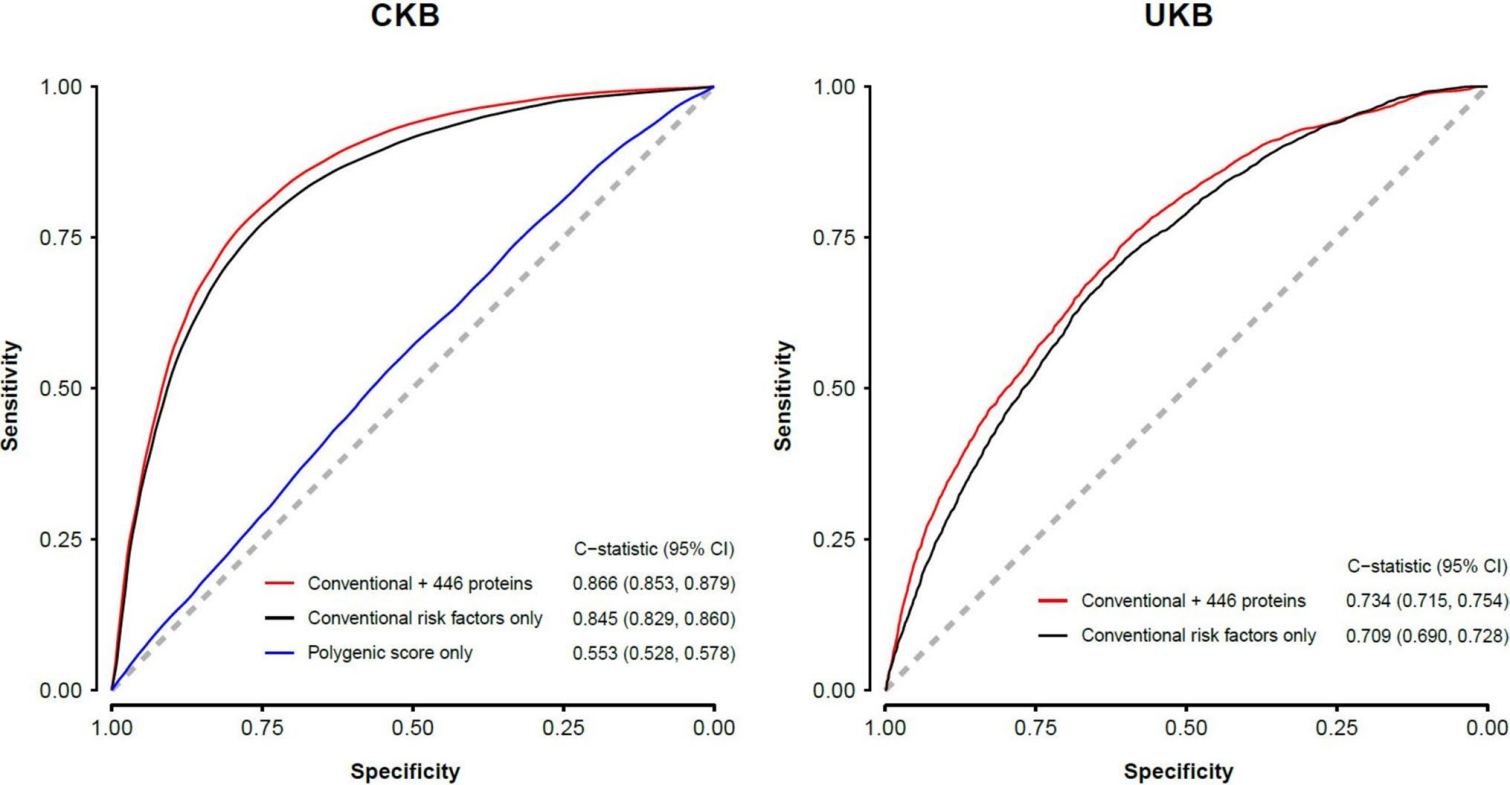
Receiver operator characteristic curves for IHD with different combinations of 446 plasma proteins, polygenic scores and conventional CVD risk factors in Chinese (CKB) and European (UKB) adults. Symbols and conventions as in Fig. 4

The addition of proteins (any of three scenarios) to conventional risk factors yielded moderate improvements in risk prediction of IHD (Fig. 4). Compared with the subset of 30 proteins identified by machine learning (ML), the addition of 446 proteins to conventional risk factors was associated with only a modest additional improvement in risk prediction of IHD (C-statistic 0.866; net reclassification index (NRI) 87.5% vs C-statistic 0.859; NRI 55.0%). In contrast, adding PS to the conventional risk factors or proteins (either of three scenarios) or to both yielded little or no additional improvement in discrimination or NRI for IHD. Figure 5 presents receiver-operating characteristic (ROC) curves and C-statistics for models using conventional risk factors or PS alone, and the combined model (446 proteins and conventional risk factors). The model incorporating 446 proteins demonstrated a higher Matthews correlation coefficient (MCC) compared to the model using only conventional risk factors (0.255 vs. 0.239). Moreover, the inclusion of these 446 proteins together with conventional risk factors further improved the MCC to 0.258. Calibration plots for these models are shown in eFigure 2. Use of conventional risk factors underestimated risk of IHD in some higher-risk categories, which were attenuated by the addition of proteins to the relevant models. Comparable results were obtained using PS that were weighted using PS obtained from European ancestry rather than East Asian populations (eFigure 3). Using only the 111 Bonferroni-significant proteins resulted in a C-statistic of 0.840 (95% CI: 0.825, 0.855). Adding these 111 proteins to conventional risk factors yielded additional improvements, with a C-statistic increasing to 0.862, and an NRI by 84.6%.

In analyses stratified by sex, there were smaller C-statistics in men than in women for conventional and PS models (0.810 vs 0.862; eFigure 4), while adding 446 proteins to conventional and genetic models yielded comparable NRI in both men and women (87.6% vs 84.4%). In analyses stratified by duration of follow-up, adding 446 proteins to conventional and genetic models also yielded comparable changes in C-statistics for shorter (i.e. < 3 years) vs longer (≥ 3 years) duration of follow-up (0.885 to 0.905 vs 0.841 to 0.860; NRI: 66.9% vs 86.6%, respectively: eFigure 5).

### External validation in UKB

Using weights derived from 446 proteins in the CKB model, we observed a C-statistics of 0.725 (0.705–0.745) for IHD in UKB. The addition of 446 proteins to conventional risk factors yielded C-statistics of 0.734 (95%CI: 0.715–0.754) and a 46.7% (38.9–54.6%) improvement in NRI for IHD (eFigure 5, eFigure 6).

## Discussion

This is the first large study to compare risk prediction of IHD associated with plasma proteins, conventional risk factors and PS in Chinese adults. We demonstrated that proteomics outperformed conventional risk factors and PS for prediction of risk of IHD when assessed alone and greatly improved risk prediction when combined with conventional models, particularly at the extremes of the absolute risk distribution (e.g. both low-risk and high-risk categories). In contrast, PS alone or in combination with conventional risk factors with or without proteomics resulted in little additional improvement in prediction of IHD. Boruta analyses identified a subset of 30 proteins that accounted for almost 90% of the improvement in NRI for IHD conferred by all 2923 proteins. Moreover, the identical proteins associated with IHD in Chinese in CKB yielded comparable, albeit somewhat more modest, improvement in risk prediction of IHD in European adults.

Recently several studies conducted in European ancestry populations have evaluated the utility of protein biomarkers for prediction of CVD risk (eTable 6)^.^ [7–13] Overall, the number of the proteins evaluated ranged from 80 to 5,000 proteins, and the number of participants from 813 to 3,525. With the exception of one study that used MI as an outcome [7], all other studies evaluated composite CVD outcomes that typically included IHD, stroke, heart failure (HF), CVD mortality or all-cause mortality. Overall, the addition of protein biomarkers to conventional risk factors yielded improvements in AUC ranging from 0.04 to 0.10 and improvements in NRI ranging from 1 to 43%, depending on the number of proteins (range: 8–80 proteins) and conventional risk factors evaluated in the models.

A previous prospective study used a gradient boosting ML methods to identify 50 proteins from a panel of 368 Olink proteins (as opposed to 30 from almost 3000 proteins in the present study) in 822 European adults to predict risk of CVD [7]. The latter study showed that these 50 proteins outperformed conventional risk factors (age, gender, BMI, SBP, smoking, T2D, total cholesterol (TC), high-density lipoprotein (HDL) and triglyceride (TG)) for prediction of myocardial infarction (MI), with AUC of 0.754 compared with 0.730. However, it did not provide any results for NRI, precluding comparisons with the present study. In another study of 5000 proteins in 813 European adults, 27 proteins yielded better IHD risk prediction in women than men (AUC: 0.81 vs 0.61) [11], consistent with the findings of the present study.

Conventional risk scores for CVD, including SCORE2 and PCE, typically yielded AUC statistics for IHD of < 0.80 in European populations, with the models evaluated including both blood-based and non-blood-based risk factors [3]. In the present study, we used non-blood based conventional risk factors that had been previously validated as the base model, which included age, sex, smoking, T2D, SBP and waist circumference (WC) [23]. This conventional model yielded C-statistics for IHD of 0.845, with no appreciable improvement by addition of ApoB/ApoA1 ratio. In a previous study of 41,271 Chinese adults, combined analyses of blood-based (TC and HDL) and non-blood-based (e.g. region, smoking and WC) risk factors yielded C-statistics of 0.756 for coronary artery disease (CAD) that included MI or occlusion of the coronary arteries [24]. The non-blood-based conventional risk factors used in CKB also performed well when validated in an external population in UKB, demonstrating their reliability and validity for risk prediction of IHD.

In the present study, the improvement in NRI for IHD associated with 446 or 30 ML-selected Olink proteins was greater than estimates reported in previous studies (eTable 6). Possible reasons for the discrepant results in NRI between the present and previous studies may reflect differences in disease outcomes evaluated (e.g. IHD vs composite outcomes involving CVD mortality or all-cause mortality in previous studies [8, 10, 11]), type of statistical models used, the methods by which NRI were calculated (category-free vs categorical), number of proteins included in the models, or the type of proteomics assay used. Moreover, differences in analytical methods (both ML and conventional risk prediction models) could also affect discrimination of IHD. Furthermore, multi-collinearity and non-linear relationships may occur in analyses involving large numbers of proteins, which may operate differently between different studies. The present study used a two-staged approach in which we first selected 446 proteins that were significantly associated with IHD in conventional analyses and subsequently used ML approaches to identify the most informative subset of proteins and validated this selection in an independent population to determine the optimum proteomics panel for risk prediction of IHD. Importantly, using only 111 Bonferroni-significant proteins yielded comparable results to those obtained with the FDR method.

PS have been widely evaluated for prediction of IHD and other major diseases, albeit such studies have been largely confined to European ancestry populations [25]. In a recent European study involving 6733 participants with 363 atherosclerotic cardiovascular disease (ASCVD) cases, the improvement in C-statistics between conventional models (SCORE2 and PCE) and those combining PS with conventional models were 0.008 and 0.007, corresponding to 9.6% and 12.0% improvement in NRI, respectively. [25] Likewise, in a study of 41,271 Chinese individuals with 1303 incident CAD cases, the addition of PS (comprising 540 single nucleotide polymorphism [SNPs]) to the conventional risk factors (age, SBP, TC, HDL, smoking, T2D, WC, region, urbanization, and family history of ASCVD) yielded a 1% improvement in C-statistic and 3.5% improvement in NRI [24]. The present study included a much larger number of SNPs (East Asians: 32,809; Europeans: 657,648), which were further weighted in independent trans-ethnic meta-analysis of European and East Asian populations to improve the construction of PS. However, the addition to PS to conventional risk factors yielded only a 0.3% (or 0.2% using Europeans weight) improvement in C-statistics for IHD, indicating little relevance of PS for risk prediction of IHD in Chinese adults compared with conventional and proteomics risk models.

The predictive accuracy of PS can vary significantly across populations due to differences in genetic architecture, environmental factors, and gene-environment interactions. For example, European populations, which have been extensively studied in GWAS, exhibit different allele frequencies compared to Chinese and other ancestry populations. Genetic variants identified in a specific population may not be as strongly associated with IHD as in other populations due to different underlying genetic architecture. Moreover, the strength of linkage disequilibrium with causal variants varies between populations, which can influence the effectiveness of PS across ethnic groups. Furthermore, IHD risk is influenced not only by genetic, but also by epigenetic factors which differ between populations, potentially modulating any genetic susceptibility to IHD. The development of population-specific PS or models that integrate genetic data from ancestry-specific populations could help address these limitations in future studies.

Although the individual proteins included in risk prediction models differed between studies, there was an overlap in the subset of 30 proteins identified by ML in the present study with those evaluated in previous studies using the Olink platforms, which included NT-proBNP, Growth Differentiation Factor 15 (GDF15) and Matrix Metallopeptidase-12(MMP12) (eTable 6). In the present study, the sole inclusion of NT-proBNP in addition to conventional CVD risk factors yielded only a modest improvement in C-statistic (0.845 to 0.851) and NRI by 2.7%. NT-proBNP is released by cardiac myocytes in response to volume expansion and pressure overload, and is a highly informative biomarker of CVD sub-types including stratification of heart failure subtypes in clinical practice. Future research could evaluate extending evaluation of NT-proBNP for early detection of subclinical CVD subtypes, optimizing treatment regimens, and monitoring responses to drug treatment for heart failure and other CVD subtypes. Inclusion of GDF15 or MMP12 together with conventional risk factors only modestly improved C-statistics from 0.845 to 0.849 and 0.845 to 0.847, respectively, consistent with previous study findings [26, 27]. GDF-15 is secreted in response to myocardial injury, and is potentially relevant to several CVD subtypes, including prediction of recurrence or death following MI. Moreover, GDF15 could also be used for early detection of CVD subtypes, particularly when combined with other established markers like troponins. The ability of GDF15 to predict outcomes in a range of CVD types make it a promising biomarker to improve risk stratification and use personalized medicine approaches in clinical practice for primary and secondary prevention. However, genetic studies have not provided any support for the causal relevance of NTproBNP or GDF15 for IHD, limiting their potential as primary protein targets for drug treatment and prevention of IHD. [16, 28]

Previous studies have demonstrated that the addition of individual plasma proteins to traditional cardiovascular risk factors yielded only marginal improvements in prediction of CVD risk. However, the present study demonstrated that the addition of 30 proteins, identified through ML approach, to conventional risk factors significantly improved risk prediction of IHD. Hence, the integration of proteomic panels into clinical practice could be more feasible with the emergence of cost-effective high-throughput proteomic assays that require minimal plasma volumes. In addition, advances in ML could facilitate application of proteomics in precision medicine approaches in clinical practice. In contrast with conventional clinical risk scores or genetic scores, proteomic scores reflect both lifestyle and genetic factors and enable a more personalized approach to primary prevention of CVD.

The chief strengths of the present study include the prospective design, large numbers of well-characterised incident IHD cases, large numbers of proteins analysed, replication of IHD prediction in European populations for an identical set of proteins identified in a Chinese population, and systematic assessment of three different types of risk prediction models for IHD. Moreover, in addition to conventional methods, we used Boruta to select a smaller subset of proteins for prediction of IHD, which may inform future applications in clinical practice. Further, we employed category-free NRI to avoid the need for arbitrary categories, thereby enhancing the interpretation and reproducibility of NRI internally and across various studies. We have also incorporated the MCC as a metric to evaluate the classification performance of our risk prediction models. The MCC metric demonstrated improvement only when the predictions achieved satisfactory results across all four categories of the confusion matrix (true positives, false negatives, true negatives, and false positives). In the present study, prediction models were internally validated in CKB, by using tenfold cross-validation to minimize the risk of over-fitting. However, the present study also had several limitations. First, despite internal validation based on participants in the subcohort, we were unable to conduct external replication in other East Asian populations due to the lack of available data. Second, no absolute levels of the proteins were provided to facilitate direct comparisons between studies. Third, there were no data available on conventional plasma lipids including LDL, HDL and TG in these 4000 CKB participants, which may affect the performance of conventional risk models, although the addition of ApoB/ApoA1 ratio did not alter prediction of IHD. Fourth, we applied a multiple testing correction (FDR) at all stages of analyses, which while appropriate could have resulted in false negative findings. To demonstrate the robustness of the study findings, we also used a Bonferroni correction, which should yield a more stringent correction than the FDR method. Lastly, since many of the included proteins have not been previously implicated in population studies of CVD subtypes, further studies are required to elucidate the mechanisms underlying their role in atherosclerosis and occlusive vascular diseases.

Overall, the present study identified novel associations of plasma proteomics with risk of IHD independent of conventional CVD risk factors in Chinese adults. Proteomics-based risk models outperformed conventional CVD risk factors and greatly exceeded PS for IHD risk prediction with a comparable performance in both Chinese and European populations. Moreover, such proteins improved detection of individuals at high-risk of IHD beyond conventional risk factors. Most of the improvements in NRI for IHD were obtained by a small subset of proteins, which should facilitate their future use in clinical practice. The findings suggest that plasma proteomics should be considered as an adjunct to conventional risk factors to enhance risk prediction of IHD in precision medicine in diverse populations.

## Supplementary Information

Below is the link to the electronic supplementary material.

Supplementary file1 (DOCX 669 KB)

## Data Availability

The China Kadoorie Biobank (CKB) is a global resource for the investigation of lifestyle, environmental, blood biochemical and genetic factors as determinants of common diseases. The CKB study group is committed to making the cohort data available to the scientific community in China, the UK and worldwide to advance knowledge about the causes, prevention and treatment of disease. Information on what data is available to open access users and how to apply for it are provided on the CKB website: http://www.ckbiobank.org/site/Data+Access. Applicants should submit a research proposal to ensure that any analysis is performed by *bona fide* researchers. Researchers who are interested in obtaining additional information or data included in this paper should contact ckbaccess@ndph.ox.ac.uk. For any data that are not currently available to open access, researchers are welcome to apply for a formal collaboration with the CKB study group.

## Code availability

Custom code used for the statistical analyses in this manuscript can be made available to external researchers to bona fide researchers upon reasonable request.
