# Supplementary material for: Risk prediction of ischemic heart disease using plasma proteomics, conventional risk factors and polygenic scores in Chinese and European adults
Source: Eur J Epidemiol. 2024 Nov 22;39(11):1229–40. doi: 10.1007/s10654-024-01168-8 (PMC11646273; doi:10.1007/s10654-024-01168-8)
Supplement: Supplementary file 1 — Supplementary file1 (DOCX 669 KB) [file 10654_2024_1168_MOESM1_ESM.docx]

Supplementary material

**“Risk prediction of ischemic heart disease using plasma proteomics, conventional risk factors and polygenic scores in Chinese and European adults”**

**Table of Contents**

[Members of the China Kadoorie Biobank Collaborative Group 2](#_Toc156811457)

[Supplementary methods 3](#_Toc156811458)

[eTable 1: Baseline characteristics of IHD cases and sub-cohort participants in CKB 7](#_Toc156811459)

[eTable 2: Number of quality control (QC) and assay warnings per assay, by OLINK batch in CKB 8](#_Toc156811460)

[eTable 3: Proportions of samples with values below level of detection (LOD) per assay, by OLINK batch in CKB 9](#_Toc156811461)

[eTable 4: Number of proteins significantly associated at FDR<0.05 with risk of IHD after exclusion of proteins with assay warning and below level of detection (LOD) in the final model*, by OLINK batch in CKB 10](#_Toc156811462)

[eTable 5: List of 29 proteins selected by Boruta machine learning in CKB 11](#_Toc156811463)

[eTable 6: Characteristics of previously published studies using different proteomic platforms for prediction of CVD risk 12](#_Toc156811464)

[eFigure 1A: Adjusted HRs for risk of IHD by quartiles of selected top 20 proteins in observational analyses of CKB participants, first OLINK batch 13](#_Toc156811465)

[eFigure 1B: Adjusted HRs for risk of IHD by quartiles of selected top 20 proteins in observational analyses of CKB participants, second OLINK batch 14](#_Toc156811466)

[eFigure 2: Calibration plots for conventional CVD risk factors, polygenic score and combined model in CKB 15](#_Toc156811467)

[eFigure 3: Comparison of predictive ability of conventional CVD risk factors, polygenic scores (Europeans weight) and plasma proteins for discrimination of IHD in Chinese adults 16](#_Toc156811468)

[eFigure 4: Comparison of predictive ability of conventional CVD risk factors, polygenic scores and proteins for discriminating risk of IHD by sex in CKB 17](#_Toc156811469)

[eFigure 5: Comparison of predictive ability of conventional CVD risk factors, polygenic scores and proteins for discriminating risk of IHD by length of follow up in CKB 18](#_Toc156811470)

[eFigure 6: Comparison of predictive ability of conventional CVD risk factors, polygenic scores and proteins for discriminating risk of IHD in CKB and UKB 19](#_Toc156811471)

### Members of the China Kadoorie Biobank Collaborative Group

**International Steering Committee:** Junshi Chen, Zhengming Chen (PI), Robert Clarke, Rory Collins, Liming Li (PI), Chen Wang, Jun Lv, Richard Peto, Robin Walters.

**International Co-ordinating Centre, Oxford:** Daniel Avery, Derrick Bennett, Ruth Boxall, Ka Hung Chan, Yiping Chen, Zhengming Chen, Johnathan Clarke, Robert Clarke, Huaidong Du, Ahmed Edris Mohamed, Hannah Fry, Simon Gilbert, Pek Kei Im, Andri Iona, Maria Kakkoura, Christiana Kartsonaki, Hubert Lam, Kuang Lin, James Liu, Mohsen Mazidi, Iona Millwood, Sam Morris, Qunhua Nie, Alfred Pozarickij, Paul Ryder, Saredo Said, Dan Schmidt, Becky Stevens, Iain Turnbull, Robin Walters, Baihan Wang, Lin Wang, Neil Wright, Ling Yang, Xiaoming Yang, Pang Yao.

**National Co-ordinating Centre, Beijing:** Xiao Han, Can Hou, Qingmei Xia, Chao Liu, Jun Lv, Pei Pei, Dianjanyi Sun, Canqing Yu

**10 Regional Co-ordinating Centres:**

**Guangxi** Provincial CDC: Naying Chen, Duo Liu, Zhenzhu Tang. Liuzhou CDC: Ningyu Chen, Qilian Jiang, Jian Lan, Mingqiang Li, Yun Liu, Fanwen Meng, Jinhuai Meng, Rong Pan, Yulu Qin, Ping Wang, Sisi Wang, Liuping Wei, Liyuan Zhou. **Gansu** Provincial CDC: Caixia Dong, Pengfei Ge, Xiaolan Ren. Maiji CDC: Zhongxiao Li, Enke Mao, Tao Wang, Hui Zhang, Xi Zhang. **Hainan** Provincial CDC: Jinyan Chen, Ximin Hu, Xiaohuan Wang. Meilan CDC: Zhendong Guo, Huimei Li, Yilei Li, Min Weng, Shukuan Wu. **Heilongjiang** Provincial CDC: Shichun Yan, Mingyuan Zou, Xue Zhou. Nangang CDC: Ziyan Guo, Quan Kang, Yanjie Li, Bo Yu, Qinai Xu. **Henan** Provincial CDC: Liang Chang, Lei Fan, Shixian Feng, Ding Zhang, Gang Zhou. Huixian CDC: Yulian Gao, Tianyou He, Pan He, Chen Hu, Huarong Sun, Xukui Zhang. **Hunan** Provincial CDC: Biyun Chen, Zhongxi Fu, Yuelong Huang, Huilin Liu, Qiaohua Xu, Li Yin. Liuyang CDC: Huajun Long, Xin Xu, Hao Zhang, Libo Zhang. **Jiangsu** Provincial CDC: Jian Su, Ran Tao, Ming Wu, Jie Yang, Jinyi Zhou, Yonglin Zhou. Suzhou CDC: Yihe Hu, Yujie Hua, Jianrong Jin, Fang Liu, Jingchao Liu, Yan Lu, Liangcai Ma, Aiyu Tang, Jun Zhang. **Qingdao** CDC: Liang Cheng, Ranran Du, Ruqin Gao, Feifei Li, Shanpeng Li, Yongmei Liu, Feng Ning, Zengchang Pang, Xiaohui Sun, Xiaocao Tian, Shaojie Wang, Yaoming Zhai, Hua Zhang, Licang CDC: Wei Hou, Silu Lv, Junzheng Wang. **Sichuan** Provincial CDC: Xiaofang Chen, Xianping Wu, Ningmei Zhang, Weiwei Zhou. Pengzhou CDC: Xiaofang Chen, Jianguo Li, Jiaqiu Liu, Guojin Luo, Qiang Sun, Xunfu Zhong. **Zhejiang** Provincial CDC: Weiwei Gong, Ruying Hu, Hao Wang,Meng Wang, Min Yu. Tongxiang CDC: Lingli Chen, Qijun Gu, Dongxia Pan，Chunmei Wang, Kaixu Xie, Xiaoyi Zhang.

# Supplementary methods

# *Proteomics assays*

# Plasma levels of 2923 proteins were measured using the Olink Explore 3072 panel in two independent batches, with 1^st^ Batch (1463 proteins) assayed in the OLINK laboratory at Uppsala, Sweden and 2^nd^ Batch (1460 proteins) assayed in OLINK laboratory at Boston, USA and each batch covered all participants and were treated independently. Stored baseline plasma samples for study participants were retrieved, thawed, and aliquoted into 96-well plates (with 8 wells per plate reserved for QC assays), with each plate containing both cases and controls in varying proportions, plated in the order that they were retrieved from storage at the Wolfson laboratory in Oxford. The results of plasma proteomics were provided using Normalized Protein eXpression (NPX) units on logarithmic (Log 2) scale.^1^

# Samples were flagged for QC warnings if incubation controls deviated by ≥0.3 from the median values for all samples on any plate and any proteins with QC warnings were excluded from subsequent analyses. Individual samples were flagged for assay warnings if values deviated by ≥3-fold from negative controls. Proteins with assay warnings or values below the LOD were included in the main analyses, but excluded in sensitivity analyses. The number of QC or sample warnings per assay and proportions with values below LOD are shown in eTables 1 and 2.

# *Statistical analyses*

# NPX data were standardized (i.e. with values divided by their SDs) and analysed as continuous variables. In observational analyses, a weighted Cox regression models were used to estimate the adjusted HRs (and 95% CIs) for IHD associated with individual proteins using the Prentice pseudo-partial likelihood for case-cohort studies.^2^ The sub-cohort participants who developed incident IHD during follow-up were censored at time of diagnosis. All analyses were stratified by sex and area (10 study areas), and adjusted initially (Model 1) for age, age^2^, time since last meal and its square, ambient temperature and its square, and plate ID, followed by additional sequential adjustments for (i) education (five categories) (Model 2); (ii) physical activity (MET-h/day) (Model 3); (iii) alcohol drinking (six categories) (Model 4); (iv) smoking (four categories) (Model 5); (v) SBP (Model 6); (vi) T2D (yes/no) (Model 7); and (vii) BMI (Model 8). For proteins significantly associated with IHD in Model 8, we further examined the shape of the associations with IHD by quartiles of individual proteins.

# *Risk prediction in CKB* Three proteomic-based risk models for IHD were constructed using: (i) all proteins assayed; (ii) proteins significantly associated with risk of IHD in *Model 8*; and (iii) a subset of proteins in proteomics-based model 2 that were identified using a Boruta machine learning algorithm which uses importance measures from multiple runs of a random forest classification algorithm to select relevant proteins for risk prediction.^3^ For construction of the PS in CKB, we sought relevant SNP data from Asian ancestry (BBJ) and European ancestry (CC4D Consortium, excluding CKB) populations that were registered in the Polygenic Score Catalogue (PS Catalog ID: PGS000337 and PGS003356) were constructed using the largest number of ancestry-specific IHD cases. After exclusion of ambiguous SNPs or SNPs with MAF<0.005 and INFO<0.3 thresholds, a total of 32,809 and 657,648 SNPs were used to construct the final PS, with the effect estimates for each SNP weighted by the BBJ and CC4D Consortium estimated effect sizes and summed across all SNPs. PS from BBJ were used for the main comparisons of IHD risk with conventional risk factors and proteomics, but PS derived from CC4D were used in sensitivity analyses. In additional sensitivity analyses, we (i) included ApoB/ApoA1 ratio together with conventional risk factors; and (ii) excluded values with assay warnings or values below the LOD. All p-values were adjusted for multiple testing by controlling the false discovery rate (FDR).^7^ For risk prediction, plasma proteins levels were centred by plate using the median of sub-cohort values. All prediction models were LASSO logistic regression models. Within each training data set, the lambda (regularisation) hyperparameter was selected by applying the one standard error rule to the AUC (i.e. largest lambda value such that AUC is within 1 standard error of the minimum) using 10-fold cross-validation.. Region-specific intercept terms were used in all models, with no penalty applied to these terms.^8^ Missing protein values were mean imputed within training data sets. Discrimination performance was measured by region-stratified C-statistics (including appropriate subject pairs as per the case-cohort design) and assessed visually by plotting ROC curves.^4^ For estimation of discrimination five repeats of 10-fold cross-validation were used (in which the study sample was split into 90% training and 10% test datasets, stratified by IHD status at the end of follow-up and ascertainment). For each cross-validation, estimated C-statistics were aggregated by their mean and standard errors were estimated as the standard deviation of the estimates divided by the square root of 10. Estimates were pooled across repeats by their mean and standard errors by the root mean square (i.e. averaged on the variance scale). ROC curves were estimated within the 10 CKB regions and each test data set were averaged to give an aggregated ROC curve. To average ROC curves, mean (sensitivity + specificity)/2 was calculated at each at each fixed (sensitivity - specificity)/2. Reclassification was measured using category-free Net Reclassification Index (NRI).^5^ To compare predicted risks between models, predicted relative risks were mean-centred. Models were trained on the whole sample 25 times (using different splits for the cross-validated selection of the regularization parameter) and for each model comparison, the median NRI was reported. Calibration was visualised by comparison of predicted and observed odds ratios. For each test data set predicted relative risks (log odds ratios) were grouped by deciles. Mean predicted log odds ratios in each group were calculated and compared to the lowest risk group, and these averaged across cross-validation folds. For each repeat of cross-validation, the observed log odds ratio in each group was calculated. Observed and predicted log odds ratios were averaged across repeat cross-validation estimates. We assessed discrimination and reclassification of IHD in combination with a previously validated conventional risk prediction model in CKB^6^ and/or newly constructed PS, both overall and by sex and duration of follow-up (<3y vs >3y). All analyses were performed used R version 4.2.2.

# *External validation of risk prediction in UK Biobank (UKB)*

# In UKB, data on an identical subset of proteins (OLINK EXPLORE) were available on 54,306 participants.^9^ After excluding individuals of non-European ancestry, prevalent CVD, or participants in pilot studies, 37,187 participants remained in the present analyses, including 630 incident cases of MI (ICD10 I21–I23). We compared risk prediction of IHD for comparable conventional risk factors and proteomics panels associated with IHD in Chinese with IHD in European population in UKB. For risk prediction in UKB, weights for proteomic risk scores were estimated from the CKB study sample using LASSO logistic regression as described above. Models were trained on the whole sample 25 times and weights were used from the model with the median estimated C-statistic. Weights were scaled to be per standard deviation in the sample. Protein risk scores were then calculated in the whole UKB sample, and used as predictors (along with conventional risk factors) in LASSO logistic regression models. Validation measures were calculated in the UKB sample using repeated cross-validation as in the internal validation.

# The reporting of the multivariable risk prediction models evaluated in this report followed the TRIPOD guidelines including scores for adherence to reporting risk prediction studies.^10,11^

# eTable 1: Baseline characteristics of IHD cases and sub-cohort participants in CKB

| **Characteristics** | **IHD cases** | **Sub-cohort** | **P-value^†^** |
| --- | --- | --- | --- |
|  | (n=1919) | (n=2004) |  |
| **Demographic and socioeconomic factors** |  |  |  |
| Mean age (SD), years | 63.9 (9.3) | 51.3 (10.5) | <0.001 |
| Female, % | 41.5 | 59.3 | <0.001 |
| Urban resident, % | 46.6 | 52.7 | 0.007 |
| ≥6 years education, % | 44.6 | 44.8 | 0.922 |
| Household income ≥35,000 yuan/year, % | 13.9 | 16.5 | 0.227 |
| **Medical history and medication, %^*^** |  |  |  |
| Hypertension | 22.6 | 12.9 | <0.001 |
| BP lowering medication | 39.6 | 32.9 |  |
| Diabetes | 9.6 | 3.9 | <0.001 |
| Medication for diabetes | 82.4 | 76.6 |  |
| **Lifestyle factors** |  |  |  |
| Mean physical activity (SD), MET-hours/day | 19.1 (10.5) | 21.3 (14.5) | <0.001 |
| Ever-regular smoker, % |  |  |  |
| Male | 78.2 | 74.5 | 0.224 |
| Female | 7.6 | 4.9 | 0.047 |
| Regular alcohol drinker, % |  |  |  |
| Male | 36.6 | 39.4 | 0.248 |
| Female | 4.8 | 3.3 | 0.606 |
| **Clinical measurements, mean (SD)** |  |  |  |
| SBP, mmHg | 139.0 (24.5) | 130.5 (21.4) | <0.001 |
| DBP, mmHg | 81.7 (12.8) | 78.0 (11.1) | <0.001 |
| BMI, kg/m² | 24.3 (3.8) | 23.9 (3.5) | <0.001 |
| WC, cm | 82.5 (10.6) | 80.3 (9.9) | <0.001 |
| RBG, mmol/L | 6.9 (3.9) | 6.0 (2.3) | <0.001 |
| Fasting time, hours | 5.2 (4.4) | 5.1 (5.0) | 0.895 |
| Abbreviations: SBP=Systolic blood pressure; DBP=Diastolic blood pressure; BMI=Body mass index; WC=Waist circumference; RBG=Random blood glucose; MET: Metabolic equivalent of task. | | | |
| Means and percentage are directly stadnardised to the age, sex and region distribution of the subcohort | | | |
| ^*^ Medical history was based on self-report of physician-diagnosed conditions, with use of medication restricted to those reporting relevant conditions. | | | |
| ^†^ t-test (from an adjusted linear regression model) for continuous characteristics. Cochran-Mantel-Haenszel chi-squared test for binary characteristics. | | | |

# eTable 2: Number of quality control (QC) and assay warnings per assay, by OLINK batch in CKB

|  | **Number of individuals with warnings per assay** | | | | | |
| --- | --- | --- | --- | --- | --- | --- |
|  | **0-49** | | **50-99** | | **100-200** | |
|  | **Assay** | **QC** | **Assay** | **QC** | **Assay** | **QC** |
| **1^st^ batch** | 1345 | 666 | 48 | 334 | 23 | 472 |
| **2^nd^ batch** | 1433 | 807 | 30 | 96 | 2 | 566 |

# eTable 3: Proportions of samples with values below level of detection (LOD) per assay, by OLINK batch in CKB

|  | **Proportions of values below LOD per assay** | | | |
| --- | --- | --- | --- | --- |
|  | **0.0 - 0.24** | **0.25 - 0.49** | **0.50 - 0.74** | **0.75 - 1.0** |
| **1^st^ batch** | 1204 | 73 | 77 | 118 |
| **2^nd^ batch** | 703 | 123 | 218 | 425 |
| Counts are the number of assays which have a proportion in that bin. | | | | |

# eTable 4: Number of proteins significantly associated at FDR<0.05 with risk of IHD after exclusion of proteins with assay warnings and below level of detection (LOD) in the final model*, by OLINK batch in CKB

| **Exclusions** | **OLINK batches** | | |
| --- | --- | --- | --- |
|  | **1^st^ batch (N = 1463)** | **2^nd^ batch (N = 1460)** | |
| **a). Assay warnings** | 363 | 86 |  |
| **b). Below LOD** | 375 | 98 | |
| **c). Both** | 376 | 98 | |
| * Adjusted for Age, age², fasting time, fasting time², ambient temperature, ambient temperature², plate ID, education, smoking, alcohol, physical activity, SBP, diabetes and BMI. | | | |

| eTable 5: List of 30 proteins selected by Boruta machine learning in CKB | | |
| --- | --- | --- |
| **OlinkID** | **Uniprot** | **Assay** |
| OID20049 | P16860 | NPPB |
| OID20079 | P62736 | ACTA2 |
| OID20125 | NTproBNP | NTproBNP |
| OID20237 | P07911 | UMOD |
| OID20251 | Q99988 | GDF15 |
| OID20254 | Q14767 | LTBP2 |
| OID20388 | P10646 | TFPI |
| OID20400 | P01034 | CST3 |
| OID20460 | O14904 | WNT9A |
| OID20541 | P12872 | MLN |
| OID20622 | Q6UXB2 | CXCL17 |
| OID20673 | P49763 | PGF |
| OID20731 | Q9NZC2 | TREM2 |
| OID20733 | O75888 | TNFSF13 |
| OID20735 | O00300 | TNFRSF11B |
| OID20871 | P07196 | NEFL |
| OID20901 | P52943 | CRIP2 |
| OID20981 | O14763 | TNFRSF10B |
| OID21068 | O76076 | CCN5 |
| OID21325 | Q86SJ6 | DSG4 |
| OID21408 | O14558 | HSPB6 |
| OID21413 | P02760 | AMBP |
| OID21422 | Q96D42 | HAVCR1 |
| OID21439 | P39900 | MMP12 |
| OID21451 | Q9HAV5 | EDA2R |
| OID21467 | P35318 | ADM |
| OID21505 | Q14508 | WFDC2 |
| OID30212 | P29536 | LMOD1 |
| OID30301 | P15502 | ELN |
| OID30778 | P02743 | APCS |

| eTable 6: Characteristics of previously published studies using different proteomic platforms for prediction of CVD risk | | | | | | | |
| --- | --- | --- | --- | --- | --- | --- | --- |
| **First author and year of publication** | **Sample size** | **Validation** | **Platform**  **(N protein)** | **Method**  **(N proteins)** | **Outcome** | **Performance** | **Overlapped with ML selected proteins in CKB** |
| Hoogeveen *et al.*, 2020 | 822 | Y | OLINK (368) | ML (50) | MI incidence | ΔAUC: 0.10  NRI: NP | NT-proBNP, GDF15, MMP12, ADM, NPPB, CST3, CXCL17, PGF |
| Nurmohamed *et al.*, 2022 | 870 | Y | OLINK (276) | ML (50) | Composite CVD | ΔAUC: 0.04  NRI: 17% | NT-proBNP, GDF15, MMP7, ADM, KIM1, BNP |
| Nowak *et al.*, 2018 | 1211 | Y | OLINK (80) | ML (35) | Composite CVD | ΔAUC: 0.08  NRI: NP | Not provided |
| Ganz *et al.*, 2016 | 938 | Y | SomaScan (1130) | ML (9) | Composite CVD | ΔAUC: 0.05  NRI: 43% | MMP12 |
| Williams *et al.*, 2022 | 813 | Y | SomaScan (5000) | ML (27) | Composite CVD | ΔAUC: 0.06  NRI: 43% | NT-proBNP, MMP12 |
| Deo *et al.*, 2023 | 2184 | Y | SomaScan (5000) | ML (32) | Composite CVD | ΔAUC: 0.05  NRI: NP | NT-proBNP, GDF15, MMP12, TNFRSF11B, HAVCR1, WFDC2 |
| Ho *et al.*, 2018 | 3523 | N | Luminex (85) | Cox (8) | Composite CVD | ΔAUC: NP  NRI: 1% | GDF15 |

# eFigure 1A: Adjusted HRs for risk of IHD by quartiles of selected top 20 proteins in observational analyses of CKB participants, first OLINK batch

Models were stratified by sex and study area, and adjusted for age, age², fasting time, fasting time^2^, ambient temperature, ambient temperature^2^, plate ID, education, smoking, alcohol consumption, physical activity, SBP, type 2 diabetes, and BMI. The black boxes are HRs, with the size inversely proportional to the variance of logHR and the vertical lines are 95% CIs.


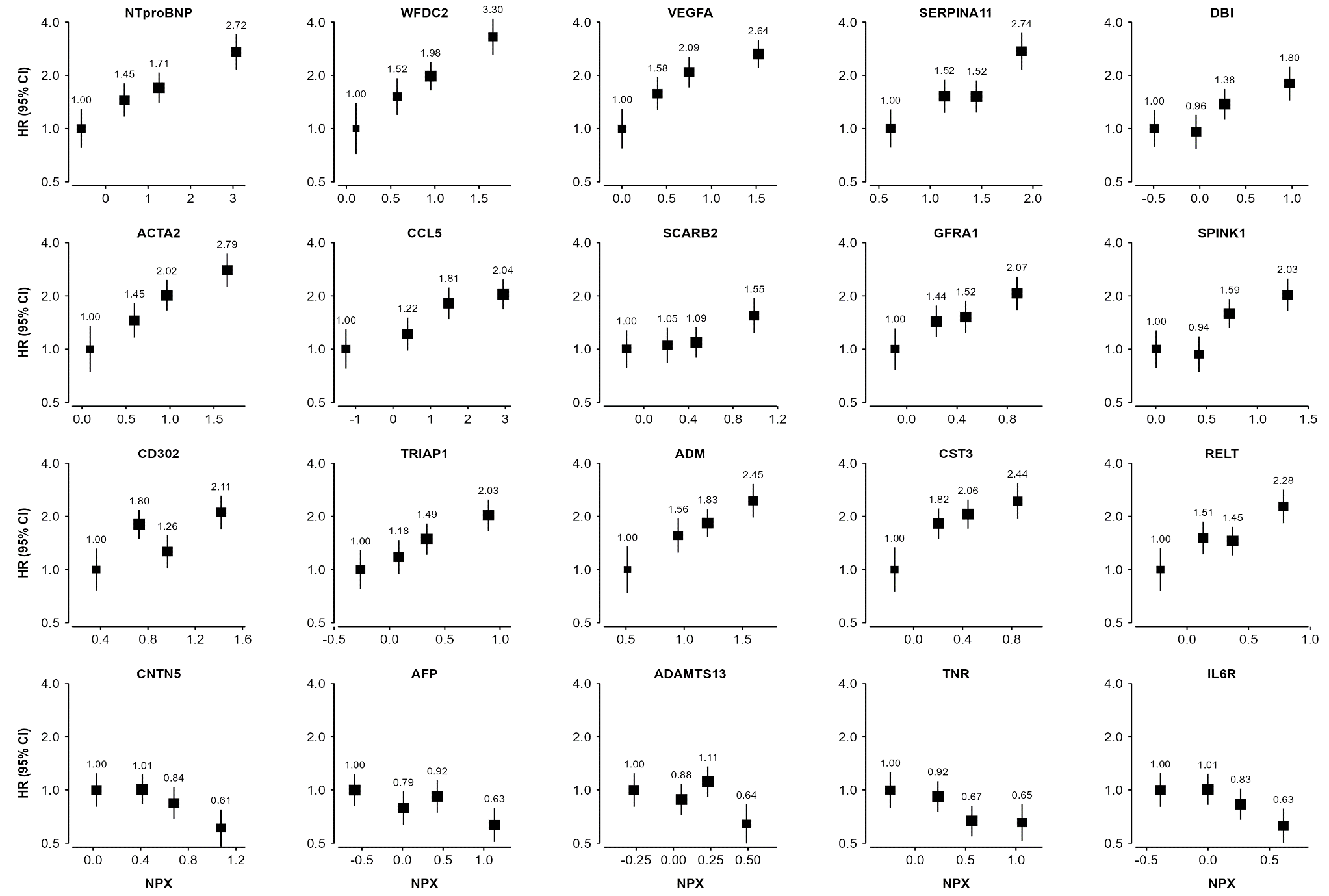


# eFigure 1B: Adjusted HRs for risk of IHD by quartiles of selected top 20 proteins in observational analyses of CKB participants, second OLINK batch

Models were stratified by sex and study area, and adjusted for age, age², fasting time, fasting time^2^, ambient temperature, ambient temperature^2^, plate ID, education, smoking, alcohol consumption, physical activity, SBP, type 2 diabetes, and BMI. The black boxes are HRs, with the size inversely proportional to the variance of logHR and the vertical lines are 95% CIs.


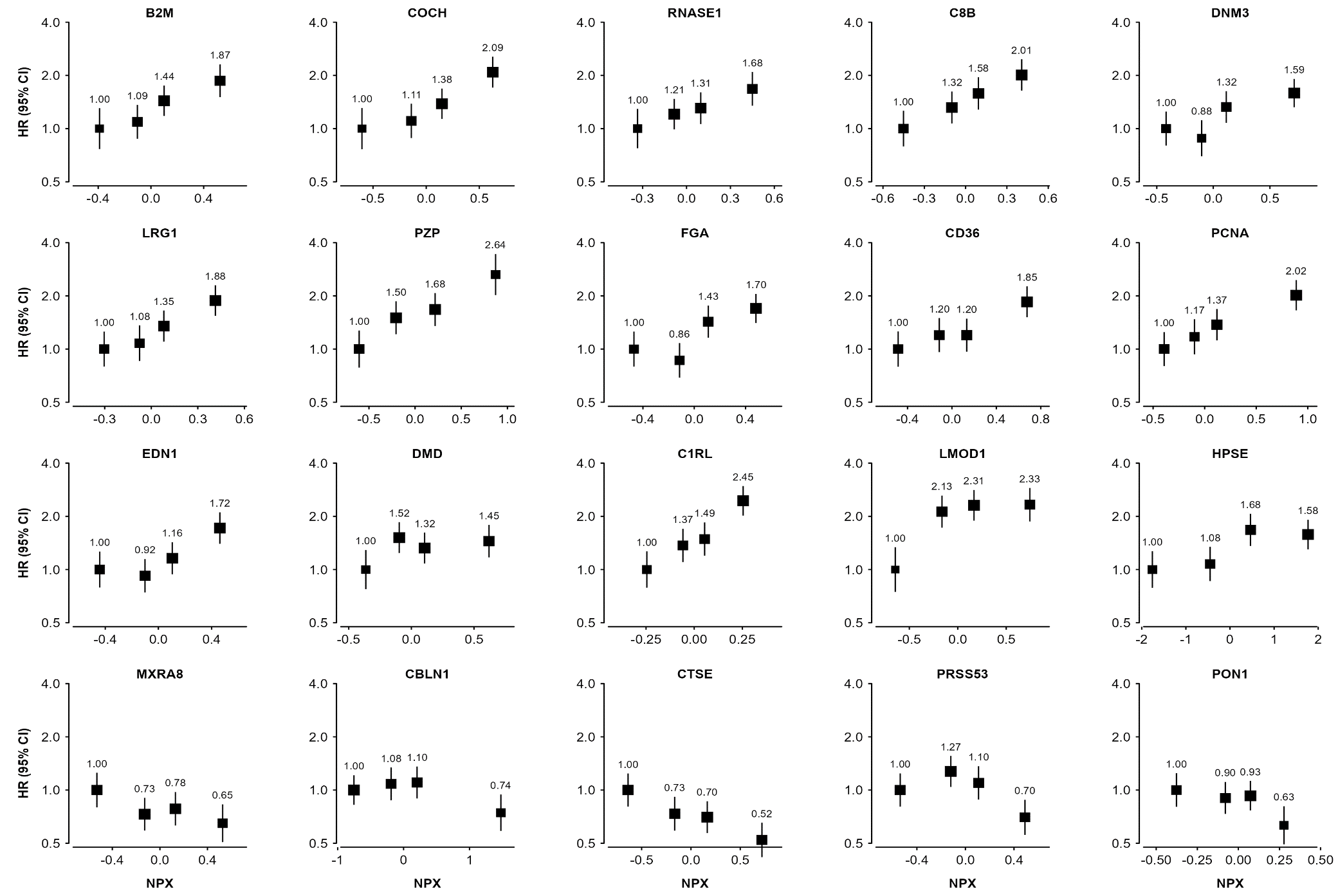


# eFigure 2: Calibration plots for conventional CVD risk factors, polygenic score and combined model in CKB

Predicted odds ratio vs. observed odds ratio per risk category deciles. Conventional risk factors include age, sex, and smoking, T2D, SBP and WC.

**
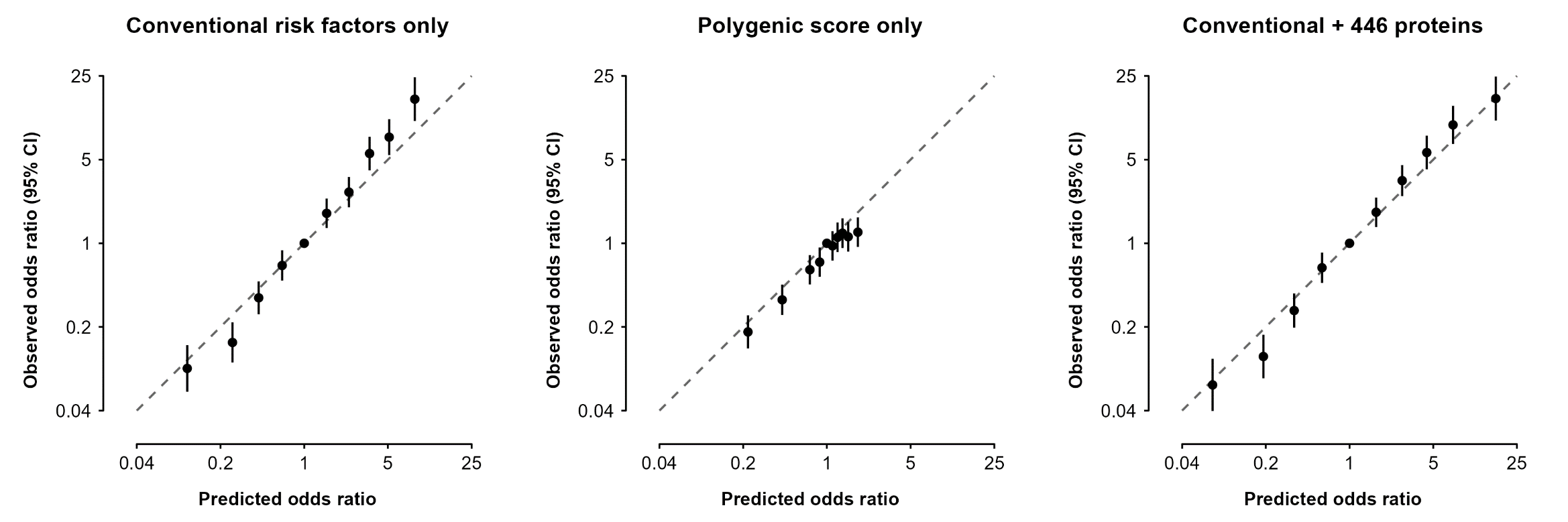
**

# eFigure 3: Comparison of predictive ability of conventional CVD risk factors, polygenic scores (Europeans weight) and plasma proteins for discrimination of IHD in Chinese adults

Conventional risk factors include age, sex, and smoking, T2D, SBP and WC. The black boxes are C-statistics, with dotted line centred at C-statistic for conventional risk model. The size of each box is inversely proportional to the variance of C-statistics and the vertical lines are 95% CIs.

#
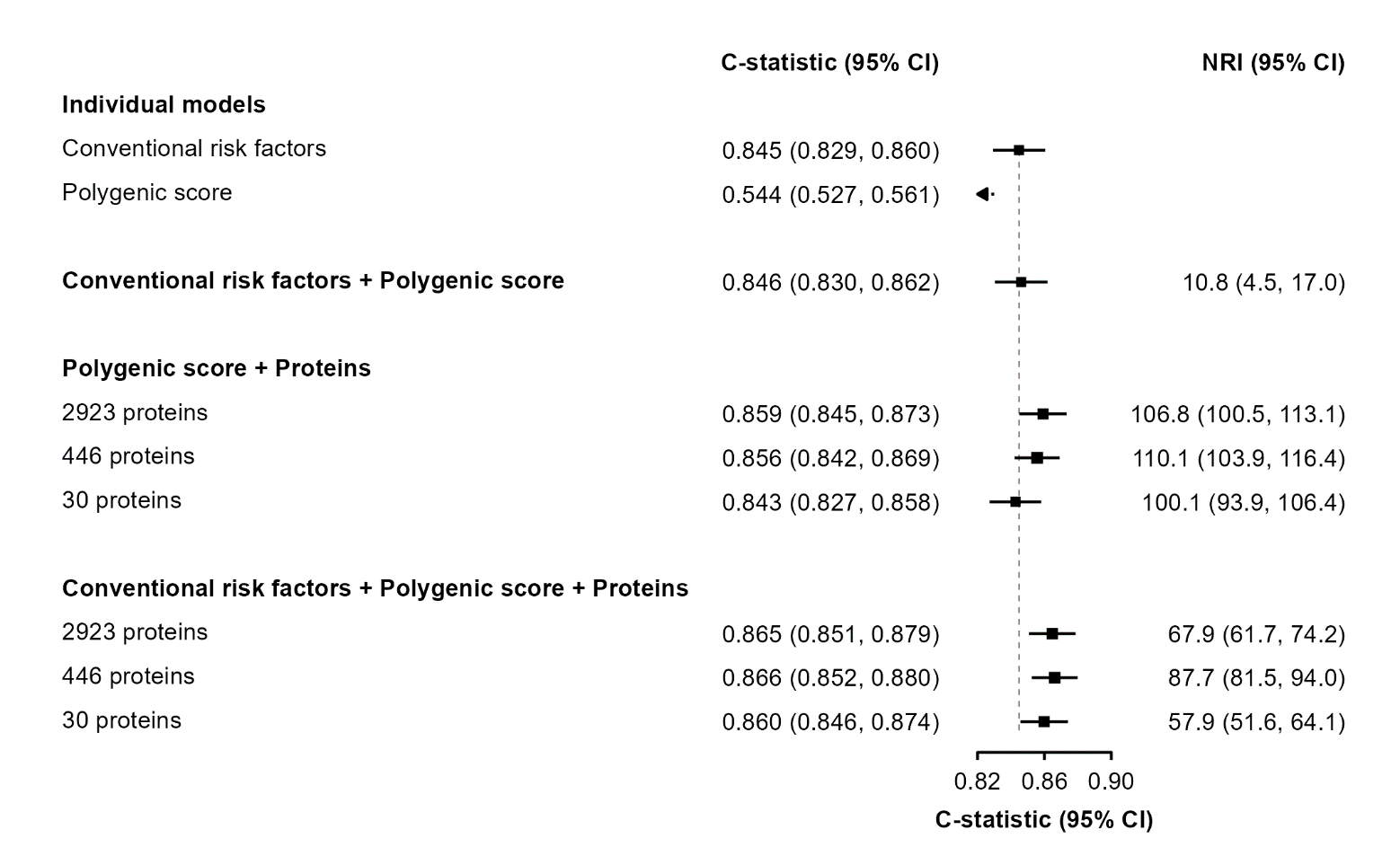


# eFigure 4: Comparison of predictive ability of conventional CVD risk factors, polygenic scores and proteins for discriminating risk of IHD by sex in CKB

Conventional risk factors include age, sex, and smoking, T2D, SBP and WC. The black boxes are C-statistics, with the dotted lines centred at C-statistic for conventional risk model. The size of each box is inversely proportional to the variance of C-statistics and the vertical lines are 95% CIs.


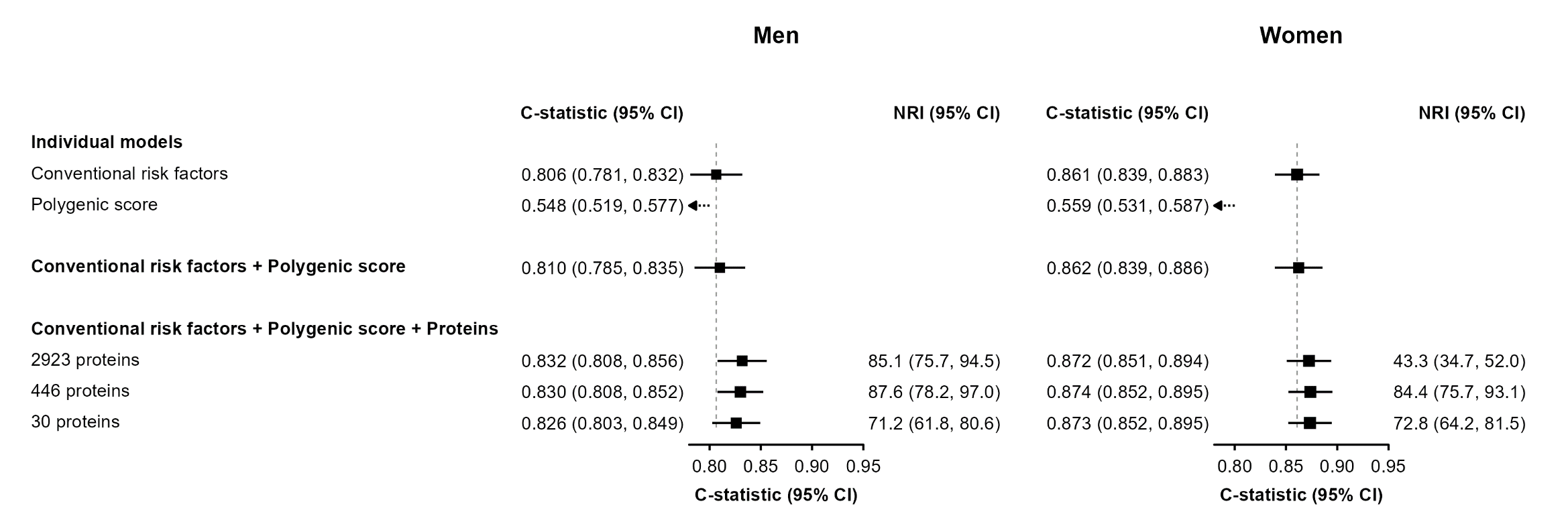


# eFigure 5: Comparison of predictive ability of conventional CVD risk factors, polygenic scores and proteins for discriminating risk of IHD by length of follow up in CKB

Conventional risk factors include age, sex, and smoking, T2D, SBP and WC. The black boxes are C-statistics, with the dotted lines centred at C-statistic for conventional risk model. The size of each box is inversely proportional to the variance of C-statistics and the vertical lines are 95% CIs.


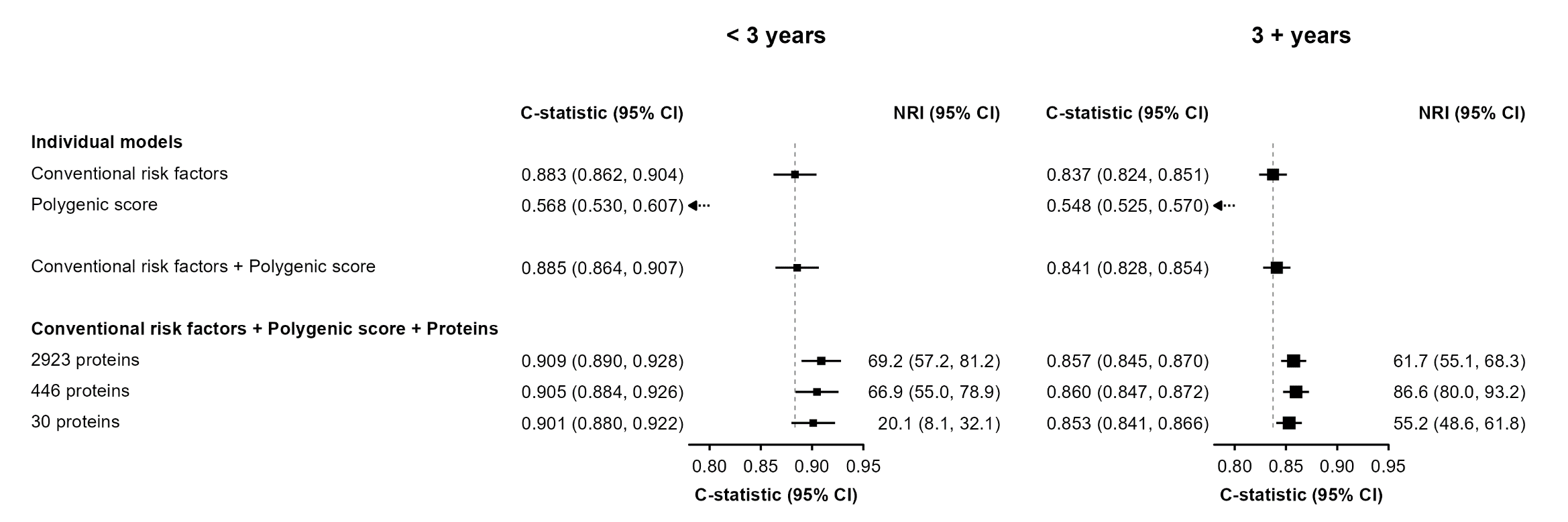


# eFigure 6: Comparison of predictive ability of conventional CVD risk factors, polygenic scores and proteins for discriminating risk of IHD in CKB and UKB

Conventional risk factors include age, sex, and smoking, T2D, SBP and WC. The black boxes are C-statistics, with the dotted lines centred at C-statistic for conventional risk model. The size of each box is inversely proportional to the variance of C-statistics and the vertical lines are 95% CIs.


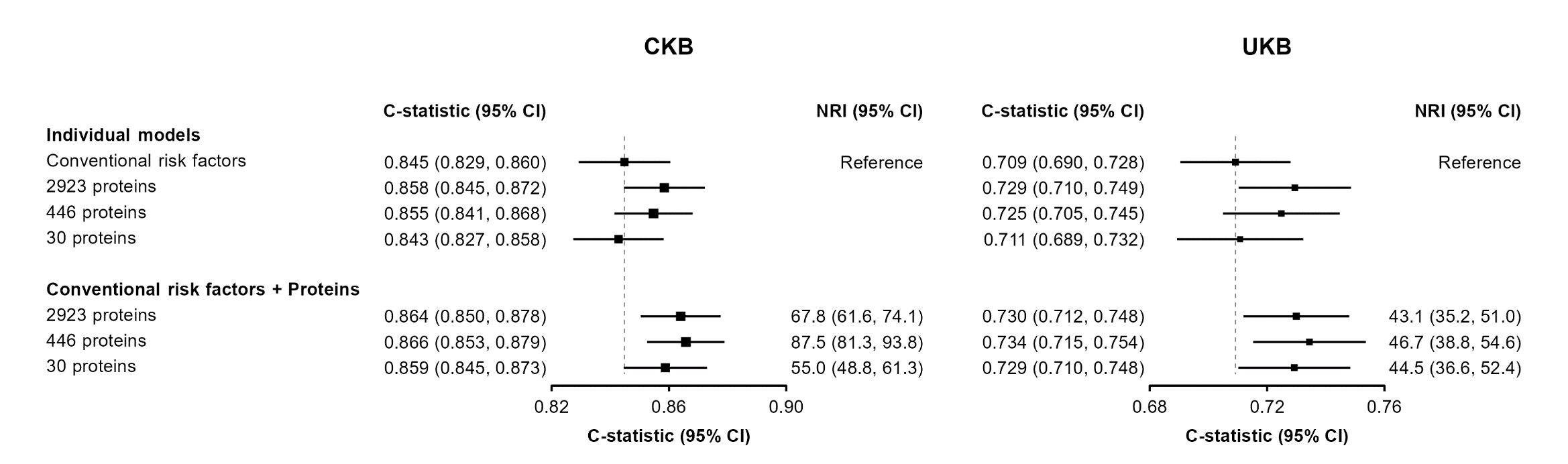


**Supplementary References**

1. Lundberg M, Thorsen SB, Assarsson E, et al. Multiplexed homogeneous proximity ligation assays for high-throughput protein biomarker research in serological material. *Mol Cell Proteomics*. Apr 2011;10(4):M110.004978. doi:10.1074/mcp.M110.004978

2. Prentice RL. A case-cohort design for epidemiologic cohort studies and disease prevention trials. *Biometrika*. 1986;73(1):1-11. doi:10.1093/biomet/73.1.1

3. Fortino V, Kinaret P, Fyhrquist N, Alenius H, Greco D. A robust and accurate method for feature selection and prioritization from multi-class OMICs data. *PLoS One*. 2014;9(9):e107801. doi:10.1371/journal.pone.0107801

4. Sanderson J, Thompson SG, White IR, Aspelund T, Pennells L. Derivation and assessment of risk prediction models using case-cohort data. *BMC Med Res Methodol*. Sep 13 2013;13:113. doi:10.1186/1471-2288-13-113

5. Pencina MJ, D'Agostino RB, Sr., Steyerberg EW. Extensions of net reclassification improvement calculations to measure usefulness of new biomarkers. *Stat Med*. 2011;30(1):11-21. doi:10.1002/sim.4085

6. Yang S, Han Y, Yu C, et al. Development of a Model to Predict 10-Year Risk of Ischemic and Hemorrhagic Stroke and Ischemic Heart Disease Using the China Kadoorie Biobank. *Neurology*. Jun 7 2022;98(23):e2307-e2317. doi:10.1212/wnl.0000000000200139

7. Benjamini Y, Hochberg Y. Controlling the False Discovery Rate: A Practical and Powerful Approach to Multiple Testing. *Journal of the Royal Statistical Society: Series B (Methodological)*. 1995/01/01 1995;57(1):289-300. doi:<https://doi.org/10.1111/j.2517-6161.1995.tb02031.x>

8. Sanderson, J., Thompson, S. G., White, I. R., Aspelund, T. & Pennells, L. Derivation and assessment of risk prediction models using case-cohort data. BMC Medical Research Methodology. doi: 10.1186/1471-2288-13-1133, 113 (2013).

9. Bycroft C, Freeman C, Petkova D, et al. The UK Biobank resource with deep phenotyping and genomic data. *Nature*. Oct 2018;562(7726):203-209. doi:10.1038/s41586-018-0579-z

10. Heus P, Damen J, Pajouheshnia R, et al. Uniformity in measuring adherence to reporting guidelines: the example of TRIPOD for assessing completeness of reporting of prediction model studies. *BMJ Open*. Apr 24 2019;9(4):e025611. doi:10.1136/bmjopen-2018-025611

11. Collins GS, Reitsma JB, Altman DG, Moons KG. Transparent Reporting of a multivariable prediction model for Individual Prognosis or Diagnosis (TRIPOD): the TRIPOD statement. *Ann Intern Med*. Jan 6 2015;162(1):55-63. doi:10.7326/m14-0697
